# Clinical characteristics of suicidal behavior in first hospitalization and drug-naïve patients with major depressive disorder

**DOI:** 10.1186/s12991-023-00484-9

**Published:** 2023-12-06

**Authors:** Xianzhi Sun, Lili Yin, Yingying Zhang, Xuebing Liu, Jun Ma

**Affiliations:** 1 Department of Psychiatry, Wuhan Mental Health Center, No. 89, Gongnongbing Road, Wuhan, Hubei China; 2 Wuhan Hospital for Psychotherapy, Wuhan, China; 3 Department of Psychiatry, People’s Hospital of Yuan’an, Yichang, China; 4 Xinyang Vocational and Technical College, Xinyang, China

**Keywords:** Suicidal behavior, Major depressive disorder, Metabolic disorder, Clinical syndrome

## Abstract

**Background:**

Major depressive disorder (MDD) is a major and common cause of suicide. The purpose of this article is to report the clinical characteristics and patterns of co-morbid suicidal behavior (SB) in first hospitalized and drug-naïve MDD patients.

**Methods:**

A total of 345 patients with first hospitalization and drug-naïve MDD with SB were included in this study, while 183 patients without SB were included as a control group. We collected socio-demographic, general clinical data and common biochemical indicators of all participants and assessed their clinical symptoms.

**Results:**

Compared to patients without SB, MDD with SB had more severe clinical symptoms and worse metabolic indicators. Duration of disease, depressive symptom scores, and thyroid stimulating hormone (TSH) levels was risk factors for SB and its number.

**Conclusions:**

MDD patients with SB suffered more severe clinical symptoms and worse metabolic indicators, and risk factors for SB in this population were identified, which may provide beneficial insight and reference for clinical prevention and intervention of SB in MDD patients.

## Introduction

Suicidal behavior (SB) is a major social and health care problem and a leading cause of death and disability worldwide [1, 2]. According to the latest data published on the official website of the World Health Organization (WHO) [3], more than 700,000 people die by suicide each year, and suicide is the fourth leading cause of death in the young age group of 15–29. The degree of variation in the reported prevalence of suicidal behavior across the globe is substantial. In some Western countries, the prevalence of suicidal ideation is reported to be 12.1–22.0%, while the prevalence of suicide attempts is reported to be 1.0–7.6% [4–8]. In contrast, reports from Japan and Korea found that the overall prevalence of suicidal ideation was 16.8–25.7% and the overall prevalence of suicide attempts was 3.9–5.4% [9, 10]. The prevalence of SB in the general population in China is relatively low, but given the large population base in China, we still cannot underestimate this social phenomenon [11].

Major depressive disorder (MDD) is a common chronic mental illness with a high disease burden, whether from personal distress, functional and relational impairment, reduced quality of life, or socioeconomic costs [12]. Whereas another well-known and notorious feature of MDD is as an independent etiology and risk factor for SB [13, 14], even in remission from depressive symptoms, people with MDD are still not immune to SB [15]. This is often attributed to dysfunction of the hypothalamic–pituitary–adrenal (HPA) axis and the hypothalamic–pituitary–thyroid (HPT) axis in patients with MDD [16, 17]. Patients with MDD diagnoses have also been reported to account for half to two-thirds of the suicidal population with mental illness [18]. The prevalence of SB in MDD inpatients is particularly high and very common [19, 20]. The main reason for this phenomenon is often attributed to the fact that inpatients with MDD tend to have more severe depressive symptoms [21], psychotic symptoms [22] and somatic symptoms [23]. Therefore, the authors believe that describing and reporting the clinical features and patterns of SB in hospitalized MDD patients is an important prerequisite for effective prevention of suicidal death in patients.

The factors influencing SB are particularly broad and complex, which involve mental illness, culture, country, environment, sociodemographic, and cultural differences [24] and the SB of the MDD diagnostic population is similarly complicated and confounded by many factors [25]. Therefore, conducting SB-related studies on first hospitalized and drug-naïve MDD patients can, on the one hand, intervene early in SB to reduce the risk of suicidal deaths and, on the other hand, minimize the influence of confounding factors on the study results. The purpose of this article is to report the clinical characteristics of SB in Chinese patients with first hospitalization and drug-naïve MDD, to report the risk factors for SB in this population, and to expect to provide useful references for clinical interventions for SB.

## Materials and methods

### Subjects

A total of 345 MDD patients who were drug-naïve and with SB were included in this study, all of whom were first hospitalized at the Wuhan Mental Health Center and the People's Hospital of Yuan'an between August 2017 and May 2023.

To be included in the study, patients had to meet the following eligibility criteria: (1). meet the diagnostic criteria for MDD according to the International Classification of Diseases, 10th Revision (ICD-10). (2). The current visit was the first treatment with no previous history of antipsychotic or antidepressant treatment. (3). Age between 18 and 60 years, (4). Chinese Han population. (5). Hamilton Depression Scale (HAMD-17) scores > 24. (6). There was a clear history of suicide attempts (SA) during the current onset cycle, and the SB occurred within 30 days prior to admission.

Patients were excluded from the study if they met any of the following criteria: (1). schizophrenia, bipolar disorder, or other types of psychiatric disorders other than MDD. (2). History of substance dependence. (3). Diagnosed with serious physical illnesses such as acute infections, tumours, cardiopulmonary dysfunctions, immune disorders, motor dysfunctions, metabolic and endocrine disorders that are being treated with medication (e.g., hypertension, hyperlipidaemia, diabetes, etc.). (4). Unable to cooperate with psycho-psychological evaluation due to serious behavioral disorders or other reasons. (5). Other forms of suicide, such as suicidal ideation, suicidal planning, etc.

Along with the collection of cases in the study group, the investigators also included a matched 183 MDD patients as a control group. The inclusion and exclusion criteria for the control group were the same as those for the study group, but the cases could not be with SB.

The study was approved by the Ethics Committee of Wuhan Mental Health Center. All participants provided written informed consent, signed by the patients themselves or their families.

### Research design

The study design was a case–control study. We first compared the differences between sociodemographic and common clinical parameters in the first hospitalized and drug-naïve MDD population with and without SB, determined the correlates influencing SB by binary logistic regression models, and finally determined the factors influencing the number of SB by multiple linear regression models.

We used a self-designed EXCLE spreadsheet to record demographic and clinical information of the target group. We completed the collection and recording of patient sociodemographic and general clinical information on the day of admission, including age, sex, height, weight, blood pressure, waist circumference (WC), onset age, duration of illness, marital status, education, etc. We focused on recording the number of suicide attempts that occurred within 30 days prior to patient admission. At the same time, we assessed patients' depressive symptoms, anxiety symptoms and severity of illness using the HAMD-17, Hamilton Anxiety Scale (HAMA-14) and Clinical Global Impression Scale—Severity of Illness (CGI-SI), respectively. In addition, we used the Positive Symptom Subscale (PSS), which is the P1–P7 items of the Positive and Negative Symptom Scale (PANSS), to assess the severity of patients' psychotic symptoms. On the second day of the patient's admission, we extracted the levels of blood lipids, fasting glucose, thyroid function, and other clinical routine tests (shown in Table 1) obtained using the patient's fasting venous blood test in the electronic medical record system.

**Table 1:** Demographic and general clinical data in different groups

Index	Study group (*n *= 345)	Control group (*n* = 183)	$t{/\chi }^{2}$	*p—*value
Age—years	36.39 ± 12.58	33.98 ± 12.57	− 2.10	0.036*
Onset age—years	34.88 ± 12.47	33.9 ± 12.53	− 0.86	0.392
Course of disease—months	13.38 ± 4.47	9.51 ± 3.2	− 11.50	<0 .001*
Gender			0.01	0.965
Male	133, 38.55%	71, 38.80%		
Female	212, 61.45%	112, 61.20%		
Marital status—(n, %)			0.02	0.879
Unmarried	109, 31.59%	59, 32.24%		
Married	236, 68.41%	124, 67.76%		
Educational background			0.89	0.345
High school and below	251, 72.75%	126, 68.85%		
Bachelor and above	94, 27.25%	57, 31.15%		
PSS	10.01 ± 3.19	7.44 ± 1.88	− 15.11	< 0.001*
HAMD	33.77 ± 2.61	30.23 ± 2.47	− 5.16	<0 .001*
HAMA	24.1 ± 3.28	20.11 ± 2.61	− 15.25	<0 .001*
CGI-SI	5.8 ± 0.69	5.34 ± 0.51	− 8.65	< 0.001*
TSH-uIU/mL	3.89 ± 2.29	1.25 ± 0.56	− 20.33	< 0.001*
FT_3_-pmol/L	4.94 ± 0.7	4.85 ± 0.69	− 1.43	0.153
FT_4_-pmol/L	16.79 ± 3.05	16.81 ± 3.15	0.07	0.946
WC-cm	79.84 ± 8.5	73.39 ± 4.78	− 11.16	< 0.001*
FBG-mmol/L	5.25 ± 0.59	4.99 ± 0.56	− 4.91	<0 .001*
TC-mmol/L	4.85 ± 0.97	4.41 ± 1.01	− 4.92	<0 .001*
TG-mmol/L	2.2 ± 1.06	1.86 ± 0.86	− 4.04	< 0.001*
LDL-C-mmol/L	2.66 ± 0.78	2.59 ± 0.78	− 1.09	0.276
HDL-C-mmol/L	1.33 ± 0.23	1.28 ± 0.21	− 2.43	0.015*
BMI-kg/m^2^	24.2 ± 1.7	23.4 ± 1.6	− 5.26	< 0.001*
SBP-mmHg	117.32 ± 10.85	113.62 ± 12.36	− 3.41	0.001
DBP-mmHg	75.05 ± 7.12	75.22 ± 7.74	0.26	0.798

*PSS* positive symptom subscale, *HAMD* Hamilton depression scale score, *HAMA* Hamilton anxiety scale Score, *CGI-SI* clinical global impression scale—severity of illness, *TSH* Thyroid stimulating hormone; *FT*_*3*_ Free triiodothyronine, *FT*_*4*_ Free tetraiodothyronine, *WC* waist circumference, *FBG* fasting blood glucose, *TC* total cholesterol, *TG* triglycerides, *LDL-C* low-density lipoprotein cholesterol, *HDL-C* high-density lipoprotein cholesterol, *BMI*: body mass index, *SBP* systolic blood pressure, *DBP* diastolic blood pressure. *p < 0.05

The investigators paid special attention to and documented whether the included population was accompanied by SB. We defined SB following the following rules: patients with SB since the onset of MDD and identified as suicide attempts were recorded by the interviewer as MDD patients with SB. The identification of such behavior, in turn, required multiple confirmations, including the patient himself, the medical history provider, and relatives living with the patient. Patients with MDD who had suicidal intent or suicidal ideation but did not commit suicide were not included in the study group.

Two psychiatrists from the medical center where the sample source was obtained who had received uniform training and had the rank of attending or above evaluated the pertinent psychological measures.

### Data analysis

The obtained normally distributed continuous variables were expressed as means and standard deviations, and the categorical variables were expressed as counts. First, the continuous variables in the two groups were compared using independent samples *t* test and the difference in the rates in the two groups was compared using Chi-square test. Second, a binary logistic regression model constructed using the variables that differed in the univariate analysis were used as independent variables and SB as the dependent variable to determine the factors influencing SB. Third, to determine the factors influencing the number of SB in the MDD diagnosis population, we constructed a multiple linear regression model using the factors influencing SB identified in the second step as independent variables and the number of SBs as the outcome variable. All *p* values were two-tailed, and the significance level was < 0.05. The statistical analyses were conducted using SPSS 27 (SPSS, Inc., Chicago, IL).

## Results

### Differences in sociodemographic and clinical parameters between the study and control groups

There were differences in a wide range of clinical parameters in the study group with SB relative to the control group without SB (Table 1). Compared to the control group, the study group had significantly higher age, duration of illness, clinical symptoms (PSS score, HAMD score, HAMA score, CGI-SI score), TSH levels, and numerous metabolic parameters (WC, FBG, TC, TG, HDL-C, BMI), all with *p* values less than 0.05.

### The factors influencing SB in MDD patients: based on a binary logistic regression model

Next, we constructed a binary logistic regression model (Backward: Wald) with SB as the outcome variable and parameters that differed in the univariate analysis as independent variables to determine the factors influencing SB (Table 2). We found that the course of disease (*B* = 0.51, *p* < 0.001, OR = 1.66, 95%CI 1.43–1.92), HAMD score (*B* = 0.45, *p* < 0.001, OR = 1.57, 95%CI 1.26–1.96), HAMA score (*B*  0.54, *p* < 0.001, OR = 1.71, 95%CI  1.38–2.11), and TSH level (B = 2.04, *p* < 0.001, OR = 7.68, 95%CI 4.34–13.59) were risk factors for SB, while TC level (B = − 0.55, *p* = 0.031, OR = 0.58, 95%CI 0.35–0.95) was a protective factor.

**Table 2:** Binary logistic regression analyses of determinants of SB in MDD patients

	Coefficients	Std. error	Wald	*p* value	95% CI for EXP (B)		
	B				Exp (B)	Lower	Upper
Constant	− 33.99	5.20	42.77				
Course of disease—months	0.51	0.08	45.38	< 0.001*	1.66	1.43	1.92
HAMD	0.45	0.11	16.10	< 0.001*	1.57	1.26	1.96
HAMA	0.54	0.11	24.36	<0 .001*	1.71	1.38	2.11
TSH—uIU/mL	2.04	0.29	48.88	<0 .001*	7.68	4.34	13.59
WC—cm	0.07	0.04	3.68	0.055	1.07	1.00	1.15
TC—mmol/L	− 0.55	0.26	4.66	0.031*	0.58	0.35	0.95
SBP—mmHg	− 0.04	0.02	3.88	0.050	0.96	0.93	1.00

*HAMD* Hamilton depression scale score, *HAMA* Hamilton anxiety scale Score, *TSH *Thyroid stimulating hormone, *WC* waist circumference, *TC* total cholesterol, *SBP* systolic blood pressure. *p < 0.05

### Factors influencing the number of SB among MDD patients: based on a multiple linear regression model

Finally, in the study group, the frequency distribution of SA was as follows: 74.49% (257/345) of patients with 1 SA since onset, 19.42% (67/345) with 2 SA, 4.93% (17/345) with 3 SA, and 1.16% (4/345) with 4 SA. We constructed a multiple linear regression model using the number of SA as the dependent variable and the parameters affecting SB identified in the previous step as independent variables to determine the factors associated with the number of SA (Table 3). The results revealed that the course of disease (*B* = 0.02, *t* = 2.46, *p* = 0.015, 95%CI 0.00–0.03), HAMD scores (*B* = 0.11, *t* = 9.10, *p* < 0.001, 95%CI 0.09–0.14), and TSH levels (*B* = 0.11, *t* = 8.43, *p* < 0.001, 95%CI  0.08–0.13) were risk factors for the number of SA, while TC levels (*B* = − 0.12, *t* = − 3.80, *p* < 0.001, 95%CI − 0.19–− 0.06) was a protective factor.

**Table 3:** Multiple linear regression analysis of the factors determining the number of SBs in MDD patients

	Coefficients	Std. error	*t*	*p *value	95% CI	
	*B*				Lower	Upper
Constant	− 2.45	0.41	− 5.91			
Course of disease—months	0.02	0.01	2.46	0.015*	0.00	0.03
HAMD	0.11	0.01	9.10	< 0.001*	0.09	0.14
HAMA	0.00	0.01	− 0.06	0.951	− 0.02	0.02
TSH—uIU/mL	0.11	0.01	8.43	< 0.001*	0.08	0.13
TC—mmol/L	− 0.12	0.03	− 3.80	<0 .001*	− 0.19	− 0.06

*HAMD* Hamilton Depression Scale score, *HAMA* Hamilton Anxiety Scale Score, *TSH* Thyroid stimulating hormone, *TC* total cholesterol. *p < 0.05

## Discussion

Our study reports the clinical characteristics of first hospitalization drug-naïve MDD patients with co-morbid SB, and identifies and determines risk factors for SB and its number of occurrences in the target population. The identification of these factors may be a predictor of SB in this population as well as a target for clinical intervention.

A previous study reported that populations with a diagnosis of MDD with SB had higher levels of metabolic-related indicators and thyroid function parameters [26]. Other studies have reported that patients with MDD combined with SB have more severe depressive symptoms, anxiety symptoms, and psychotic symptoms [27, 28]. These reports are highly consistent with the results of our study. More in-depth and further studies have also reported that patients with MDD combined with SB have brain dynamics abnormalities [29], poorer language academic abilities [30], more complex and difficult treatment difficulties [31], and higher socioeconomic burden [32]. Therefore, whether SB is a cause or a consequence of the above-mentioned terrible situation of MDD patients, it is reasonable to be sure that no amount of heightened attention can be devoted to MDD patients with SB.

It is well-known that HPT axis dysfunction plays an important role in SB in patients with MDD [17, 33, 34]. Several previous studies have long reported TSH as a risk factor for SB in MDD population [26, 35], even for some specific subtypes of MDD [36, 37]. In addition, TSH levels are considered to be an important predictor of severe anxiety symptoms [38] and depressive symptoms [39, 40] in patients with MDD. For this reason, it has been suggested that TSH is pivotal in many SB risks and is a central factor in the development of SB in patients with MDD [41]. Meanwhile, depressive symptoms and severity of anxiety symptoms are two other risk factors for SB for which there is consensus [42, 43]. In our study, we found that both depressive symptoms, anxiety symptoms and TSH together were identified as risk factors for SB in the MDD population. Therefore, we hypothesized that elevated TSH levels may increase the risk of suicide in MDD by increasing the severity of anxiety and depressive symptoms in MDD patients through some unexplained mechanism.

Engaging in SB is undoubtedly the highest risk of suicide other than completed suicide [44], and repeatedly committing suicide multiple times undoubtedly increases the risk of death by suicide. In the present study, we further identified factors influencing the number of SBs in the target population, and we found that: longer duration of illness, severity of depressive symptoms and higher TSH levels were all risk factors contributing to multiple SBs committed by patients, while TC levels was a protective factor. However, published studies appear to have few reports on risk factors for the number of SBs in patients with MDD. A small sample study from scholars in the Netherlands reported that persistent MDD was significantly associated with multiple suicidal ideations [45]. These are an important corroboration of our report of the duration of illness and severity of depressive symptoms as contributing factors to multiple suicides. Additional study reports a broader range of risk factors for recurrent SB compared to single SB, including female, concomitant anxiety symptoms, substance abuse, and irritability [46]. Regarding our finding of protective factors for SB, a prospective study and a case–control study of MDD populations with a history of suicide attempts, respectively, found that lower levels of TC predicted repeat suicides [47, 48], yet, some studies seem to confirm that there is no association between the two [49, 50]. The results we obtained differ from all of the above studies, which further adds to the controversy over the predictive ability of TC levels for SB. This may be due to differences in the recruitment of psychiatric samples and the different methodologies used in existing studies. In conclusion, the risk factors for multiple SBs in MDD patients may be more complex than what has been reported, and this needs to be validated by more rigorous and large prospective studies.

There are some shortcomings in our study. First, compared to the sample size of MDD patients with SB, the sample size of MDD without SB was more prevalent and easier to collect, but the sample size of MDD without SB in this study was smaller, which may have affected the general representation of each target parameter in the control group. Second, the study and control groups differed in demographic characteristics such as age and duration of disease, which may have increased the confounding factors for differences in metabolic parameters between the two groups. Third, this was a case–control study and could not explain the findings in depth in terms of causality, implying that further studies are needed to validate our speculations.

In conclusion, MDD patients with SB had more severe clinical symptoms and worse metabolic indexes. The identification of risk factors for SB and its frequency, such as disease duration, depressive symptoms, and TSH levels, may help to provide a clinical prediction of SB and a potential target for intervention in this behavior.

## Data Availability

The data sets used and/or analyzed during the current study available from the corresponding author on reasonable request.

## Abbreviations

SB: Suicidal behavior
WHO: World Health Organization
MDD: Major depressive disorder
HPA: Hypothalamic–pituitary–adrenal
HPT: Hypothalamic–pituitary–thyroid
ICD-10: The International classification of diseases 10th revision
HAMD-17: Hamilton depression scale
SA: Suicide attempt
HAMA-14: Hamilton anxiety scale
CGI-SI: Clinical Global Impression Scale—Severity of Illness
PSS: Positive symptom subscale
PANSS: Positive and negative symptom scale
TSH: Thyroid stimulating hormone
FT3: Free triiodothyronine
FT4: Free tetraiodothyronine
WC: Waist circumference
FBG: Fasting blood glucose
TC: Total cholesterol
TG: Triglycerides
LDL-C: Low-density lipoprotein cholesterol
HDL-C: High-density lipoprotein cholesterol
BMI: Body mass index
SBP: Systolic blood pressure
DBP: Diastolic blood pressure
